# Mitochondrial dysfunction and liver disease: role, relevance, and potential for therapeutic modulation

**DOI:** 10.1177/17562848211031394

**Published:** 2021-07-27

**Authors:** Paul Middleton, Nikhil Vergis

**Affiliations:** Department of Metabolism, Digestion and Reproduction, Imperial College London, St Mary’s Hospital, Praed Street, London, SW7 2AZ, UK; Department of Metabolism, Digestion and Reproduction, Imperial College London, London, UK

**Keywords:** alcohol related liver disease (ALD), hepatitis B, hepatocellular carcinoma (HCC), liver disease, mitochondria, non-alcoholic fatty liver disease (NAFLD)

## Abstract

Mitochondria are key organelles involved in energy production as well as numerous metabolic processes. There is a growing interest in the role of mitochondrial dysfunction in the pathogenesis of common chronic diseases as well as in cancer development. This review will examine the role mitochondria play in the pathophysiology of common liver diseases, including alcohol-related liver disease, non-alcoholic fatty liver disease, chronic hepatitis B and hepatocellular carcinoma. Mitochondrial dysfunction is described widely in the literature in studies examining patient tissue and in disease models. Despite significant differences in pathophysiology between chronic liver diseases, common mitochondrial defects are described, including increased mitochondrial reactive oxygen species production and impaired oxidative phosphorylation. We review the current literature on mitochondrial-targeted therapies, which have the potential to open new therapeutic avenues in the management of patients with chronic liver disease.

## Introduction

Mitochondria are key organelles involved in energy production as well as numerous metabolic processes. There is a growing interest in the role of mitochondrial dysfunction in the pathogenesis of common chronic diseases as well as in cancer development. This review will examine the role mitochondria play in the pathophysiology of alcohol-related liver disease (ARLD), non-alcoholic fatty liver disease (NAFLD), chronic hepatitis B and hepatocellular carcinoma and discuss candidates for mitochondrially targeted therapies. These conditions were chosen as they represent a large proportion of the burden of chronic liver disease and there remains a strong clinical need for new effective therapies.

### The mitochondria

**Figure 1.**
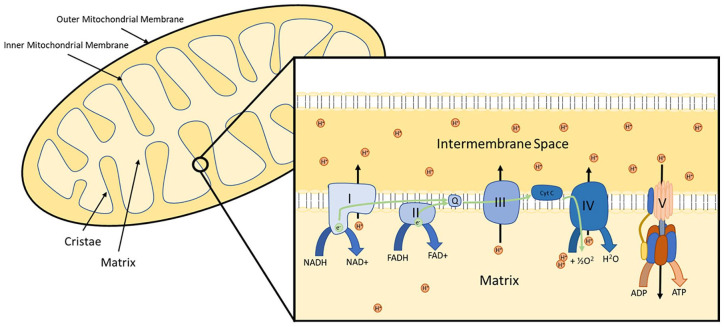
The mitochondrion and electron transport chain. Electrons are received by the electron transport chain from electron carriers (NADH and FADH). The movement of electrons through complexes I–IV allows the translocation of hydrogen ions from the mitochondrial matrix to the intermembrane space, creating an electrochemical gradient. This gradient can be released through complex V (ATP synthase), which utilises the electrochemical gradient to convert ADP to ATP.

Mitochondria originate from an engulfed alpha-proteobacterium 2 billion years ago and have since become a near-universal feature of eukaryote cells.^1^ Structurally they have an inner and outer membrane that encloses an intermembrane space.^1^ Within the inner membrane is the mitochondrial matrix where vital metabolic processes such as the tricarboxylic acid (TCA) cycle occur.^1^ The inner membrane has multiple invaginations termed cristae that contain the five complexes (I–V) of the electron transport chain (ETC). These are vital for the mitochondria’s main function of producing ATP *via* oxidative phosphorylation (Figure 1).^1^

Metabolism of energy substrates generate NADH and FADH2, which donate electrons to the ETC.^2^ Movement of electrons through the ETC induces transfer of protons across the inner membrane into the intermembrane space to create an electrochemical gradient also referred to as mitochondrial polarisation.^2^ A by-product of this process is the production of reactive oxygen species (ROS), which during normal physiology has cell-signalling functions.^2^ The electrochemical gradient is released *via* ATP synthase (complex V), which is a turbine-like complex that converts the energy from proton movement into the phosphorylation of ADP to ATP (Figure 1).^2^ In addition to ATP production, mitochondria also have roles in calcium storage and homeostasis, energy homeostasis signalling, innate immune signalling and cell apoptosis.^2^

Mitochondria are dynamic organelles that can fuse together and fissure apart under the control of fission proteins (Drp1) and fusion proteins (Mfn1/Mfn2/Opa1).^2^ Mitochondrial dynamics have key roles in mitochondrial physiology. Mitochondrial fission allows transport of the mitochondria to different cell locations as well as isolation of damaged mitochondria to allow trafficking for proteasomal degradation in a process termed mitophagy.^2^ Fusion allows the transfer of mitochondrial proteins and mitochondrial DNA (mtDNA) within the newly fused mitochondria and can therefore act as a repair mechanism.^1,2^ Mitochondria retain their own circular genome, which encodes 13 proteins, including subunits of the ETC complexes.^1^ This genome retains features of its bacterial origin, including having multiple copies within each mitochondrion.^1^

## Alcohol-related liver disease

### Mitochondrial dysfunction in alcohol-related liver disease

**Figure 2.**
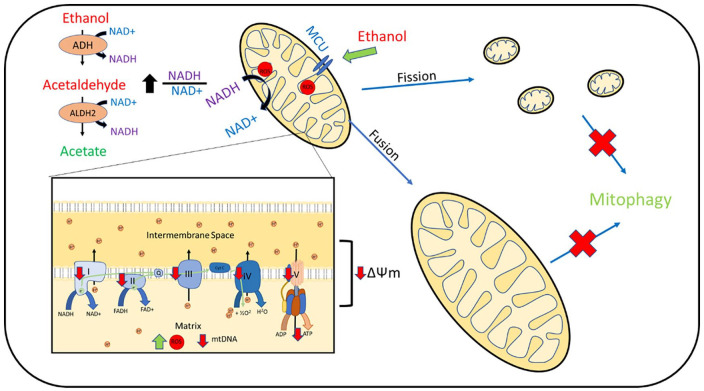
Hepatocyte mitochondrial dysfunction reported in alcohol-related liver disease models and human tissue. Ethanol is converted to acetaldehyde *via* ADH, then *via* mitochondrial ALDH2 to acetate. This process reduces NAD^+^ to NADH and increases the NADH/NAD^+^ ratio within the cell. NADH is oxidised to NAD^+^ within the mitochondrial electron transport chain. Alcohol boluses are associated with increase mitochondrial ROS production, which is associated with mtDNA damage. Impaired complex I–V function is reported with reduction in mitochondrial polarisation (ΔΨm) and ATP production. Alcohol exposure is associated with upregulation of MCU and increased mitochondrial calcium uptake. Increased mitochondrial fission is reported as well as increased fusion resulting in large megamitochondrion. Upregulation of mitochondrial fusion is associated with a more favourable outcome. Removal of damaged mitochondria *via* mitophagy is impaired. ADH, alcohol dehydrogenase; ALDH2, acetaldehyde dehydrogenase 2; MCU, mitochondrial calcium uniporter; mtDNA, mitochondrial DNA; ROS, reactive oxygen species.

Mitochondria play a key role in alcohol metabolism. Alcohol is metabolised to acetaldehyde *via* alcohol dehydrogenase (ADH), CYP2E1 and catalase.^3^ Acetaldehyde is then detoxified to acetate *via* mitochondrial acetaldehyde dehydrogenase 2 (ALDH2). ADH and ALDH2 both require NAD^+^ to act as a hydrogen acceptor in the oxidation of alcohol and acetaldehyde, respectively. In conditions of increased alcohol exposure, the cell ratio of NADH/NAD^+^ is increased (Figure 2).^3^ NADH must be recycled to NAD^+^ to allow ongoing metabolism of alcohol and acetaldehyde.^3^ This occurs through electron donation to the ETC. Hepatocyte mitochondria are found to respond to acute exposure to alcohol with an adaptive metabolic response termed the swift increase in alcohol metabolism, which shifts mitochondria function from oxidation phosphorylation to rapid cycling of NADH to NAD^+^ to facilitate alcohol and acetaldehyde metabolism.^4^ This is shown by an increase in oxygen consumption with a reduction in ATP production.^4,5^ Alcohol-stimulated increase in mitochondrial metabolism can lead to mitochondrial stress and dysfunction.

#### Alcohol-induced mitochondrial ROS and impaired mtDNA homeostasis

Repeated alcohol boluses in mice leads to increased ROS production and is associated with increased mtDNA damage and depletion.^6,7^ Upregulation of the antioxidant mitochondrial enzyme MnSOD was able to prevent mtDNA damage.^6^ Although mtDNA recovered in wild-type mice, mice with impaired MnSOD expression had persistent mtDNA defects.^6^

Chronic alcohol feeding in rat models is associated with reduced mtDNA.^8^ mtDNA damage is recognised in explant livers from patients with alcoholic hepatitis as well as alcohol-related cirrhosis.^9,10^ In addition, chronic alcohol feeding in rats is associated with impaired mitochondrial ribosome formation and function.^11–13^ Impaired mtDNA and mtDNA transcription will likely lead to defects in expression of mitochondrially encoded ETC subunits, leading to further mitochondrial dysfunction and ROS production.

#### Impaired oxidative phosphorylation

Mitochondrial function in cell culture and animal models of chronic alcohol exposure have shown significant impairments in mitochondrial function. Several animal studies have shown chronic alcohol feeding in rats is associated with reduced hepatocyte mitochondrial respiration.^14,15^ Chronic alcohol feeding is found to reduce expression and activity of mitochondrial complex I, III, IV and ATP synthase.^16–18^ In addition, both acute and chronic alcohol feeding in mice induces hepatocyte mitochondrial depolarisation.^19,20^ Similarly, mitochondrial depolarization is reported in the human hepatocyte LO2 cell line with prolonged exposure to alcohol.^21^ In keeping with impaired electron transport, mitochondrial respiration, and membrane polarization, chronic alcohol exposure is associated with reduced hepatocyte ATP production.^8,11^

#### Altered mitochondrial structure and dynamics

Chronic alcohol exposure is associated with mitochondrial structural abnormalities with alterations in mitochondrial dynamics. Hepatocyte mitochondrial swelling and impaired cristae formation is noted in rat and mouse models.^19,21^ Exposure of alcohol to hepatocyte cell lines result in initial hyper-fragmentation of mitochondrial networks.^22^ Hepatocytes from chronic alcohol-fed rats show reduced interconnectivity.^23^ Increased mitochondrial translocation of fission proteins (Drp1) and impaired expression of mitochondrial fusion proteins (Mfn1) is identified in mouse models of chronic alcohol exposure and is associated with impaired mitochondrial function.^24^ In humans, liver tissue in patients with severe alcoholic hepatitis show upregulation of Drp1 transcription, which is directly correlated to AST.^25^ However, prolonged alcohol exposure in hepatocyte cell lines led to reduced activation of mitochondrial fission protein Drp1, leading to the development of large mitochondria, termed megamitochondria.^22^ Induction of megamitochondria formation in mice models using Drp1 inactivation showed it to be a beneficial adaptive response with reduced alcohol-induced hepatoxicity.^22^ Megamitochondria are recognised in human liver biopsies of patients with alcoholic hepatitis and are associated with less severe liver dysfunction and better survival.^26,27^

#### Calcium homeostasis

The mitochondria’s role in calcium homeostasis is also affected by alcohol exposure. Chronic alcohol exposure in rats and mice has been found to increase mitochondrial calcium concentration.^19,24^ Increased expression of mitochondrial calcium uniporter (MCU) was seen in chronic alcohol-fed rat hepatocytes with an associated increased mitochondrial calcium uptake.^28^ Increased mitochondrial calcium uptake was associated with increased mitochondrial ROS production.^28^ Increased MCU expression in hepatoma cell lines has been reported to increase mitochondrial calcium concentration and promote the activity of mitochondrial matrix enzymes α-ketoglutarate dehydrogenase and isocitrate dehydrogenase, leading to a reduction in NAD^+^/NADH ratio. This inhibits the deacetylation activity of NAD^+^-dependant SIRT3, leading to reduced activity of the antioxidant enzyme superoxide dismutase 2, thereby increasing mitochondrial ROS (mtROS).^29^

#### Mitochondrial dysfunction-induced mtROS

Dysfunctional electron transport and mitochondrial dynamics can lead to further increase in mtROS production. Chronic alcohol-fed rat hepatocytes show increased mtROS production.^30,31^ In tandem with increasing oxidative stress, alcohol exposure is also found to impair antioxidant defences. Alcohol feeding in rats is associated with reduced mitochondrial glutathione concentration and impaired mitochondrial glutathione uptake.^32^ In the human hepatoma HepG2 cell line, acetaldehyde is found to stimulate mitochondrial membrane cholesterol accumulation thereby reducing the permeability of the mitochondria to glutathione uptake.^33^

#### Impaired mitophagy

Mitochondrial damage is repaired *via* mitochondrial fusion or through mitophagy. Alcohol feeding in mice is shown to reduce mitophagy.^24^ Parkin is a E3 ubiquitin ligase involved in tagging and trafficking organelles, including the mitochondria, for autophagy. Impairing mitophagy by Parkin knockout was found to promote alcohol-induced mitochondrial damage and liver injury.^34^ Alcohol is shown to inhibit mitophagy in mouse models *via* NR4A1 (nuclear receptor subfamily 4 group A member 1) mediated DNA-PKcs (DNA dependant protein kinase catalytic subunit) activation of p53. Blocking this pathway with DNA-PKcs knockout and NR4A1 knockdown models is shown to improve mitophagy, mitochondrial function and reduce alcohol-associated steatosis, fibrosis, and mitochondrial-associated apoptosis.^24^

### Role of mitochondrial dysfunction in ARLD pathogenesis

#### Inhibition of acetaldehyde metabolism

Acetaldehyde is a toxic intermediary of alcohol metabolism and can induce mitochondrial dysfunction.^20,35,36^ Mitochondrial ALDH2 is inhibited by mitochondrial oxidative damage, leading to impaired acetaldehyde metabolism and further mitochondrial damage. Alcohol feeding in mice is associated with increased ALDH2 nitrosylation and formation of peroxidation adducts with associated reduction in ALDH2 activity.^37,38^ MitoQ, a mitochondrially targeted antioxidant, was able to rescue ALDH2 function in alcohol-fed mice hepatocytes.^37^

#### Cell death

Mitochondrial damage and dysfunction promote cell death. Alcohol feeding in mice and rats is associated with increased hepatocyte apoptosis.^14,20,24^ Cytochrome c is a protein found within the inner mitochondrial membrane bound to cardiolipin.^24^ When released into the cytoplasm it leads to activation of a caspase cascade, triggering apoptosis. Alcohol-induced mtROS causes cardiolipin oxidation, thereby impairing cytochrome c binding.^24^ Cytochrome c is released from the mitochondria *via* the mitochondrial permeability transition pore (MPTP). Alcohol feeding promotes MPTP opening in rat and mouse models.^19,24^ Alcohol-mediated mitochondria calcium loading is associated with increased sensitivity to calcium induced MPTP opening.^14^ Alcohol also increases MPTP sensitivity to the pro-apoptotic protein Bax.^39^ Increased cardiolipin oxidation and MPTP opening thereby leads to increased cytoplasmic cytochrome c in alcohol-fed mouse models and increased hepatocyte cell death.^24^

#### Promotion of liver inflammation

Liver inflammation plays a key role in the pathophysiology of ARLD. Mitochondrial dysfunction has been found to promote liver inflammation. mtDNA retain features of its bacterial heritage and can therefore stimulate innate immune response. Alcohol in mouse models is found to promote the production of hepatocyte-derived extracellular vesicles containing increased quantities of mtDNA.^40^ This stimulates Kupffer cell interleukin (IL)-1b and IL-23 production in a TLR3-dependant manner.^40^ Inhibiting alcohol-induced mitochondrial fission and promoting mitophagy *via* DNA-PKcs knockout in mouse models was associated with reduced liver IL-1b and tumour necrosis factor (TNF)-α suggesting mitochondrial dysfunction plays a direct role in promoting liver inflammation.^24^ Additionally, similar to findings in hepatocytes, alcohol has direct effects on mouse macrophage mitochondria with impaired polarisation and increased ROS production. These mitochondrial impairments were associated with IL-1b hypersecretion.^41^

### Mitochondria-targeted treatments in ARLD

mtROS is a key component in mediation of alcohol-associated mitochondrial dysfunction. Several studies have shown a beneficial effect of mitochondrially targeted antioxidants in ARLD animal models. MitoQ, a mitochondrially targeted derivative of coenzyme Q10, treatment in mice is found to reduce oxidative stress, restore hepatic glutathione and promote acetaldehyde metabolism.^37^ MitoQ is associated with reduced transaminitis, hepatic steatosis, liver peroxidation and inflammation.^37^ In rat models MitoQ improved liver peroxidation and steatosis.^17^ In mouse macrophages mitoQ improved alcohol-induced IL-1b hypersecretion.^41^ Similarly, 4-OH-TEMPO (4-hydroxy-(2,2,6,6-tetramethylpiperidin-1-yl)oxyl), is an antioxidant that localises to the mitochondria and is found to improve liver peroxidation and reduce depletion of components of complex III and IV, although did not improve mitochondrial respiration.^42^ However there have been no clinical trials examining mitochondrially targeted antioxidants in ARLD. One small study of s-adenosyl-l-methionine, a precursor in glutathione synthesis, showed no efficacy compared with placebo over 24 weeks in patients with ARLD cirrhosis.^43^ *N*-acetylcysteine in addition to prednisolone in alcoholic hepatitis was found to have a short-term mortality benefit and reduced infections.^44^

## Non-alcoholic fatty liver disease

### Mitochondrial dysfunction in NAFLD

NAFLD is characterised by fat accumulation in the liver leading to inflammation, fibrosis and eventual cirrhosis.^45^ Hepatic steatosis results from insulin resistance mediated increase in adipose lipolysis, increased hepatic free fatty acid (FFA) uptake and increased hepatic *de novo* lipogenesis.^45^ Insulin resistance also results in increased hepatic gluconeogenesis.^45^ Mitochondrial β-oxidation utilises FFA to generate acetyl-CoA, which is then metabolised *via* the TCA cycle and oxidative phosphorylation as well as providing substrate for gluconeogenesis.^46^ Hepatocytes respond metabolically to this increase in energy substrates with increased mitochondrial metabolism. Patients with increased intrahepatic triglyceride content have an associated increase in TCA cycle flux as measured with ^2^H and ^13^C tracers.^46^ Increased hepatic mitochondrial β-oxidation and TCA cycle activity was identified in liver tissues of obese patients with and without NAFLD compared with lean control using high-resolution respirometry.^47^ Similarly, increased mitochondrial fatty acid oxidation was identified in animal models of obesity (ob/ob mice).^48^

#### NAFLD-induced mtROS

**Figure 3.**
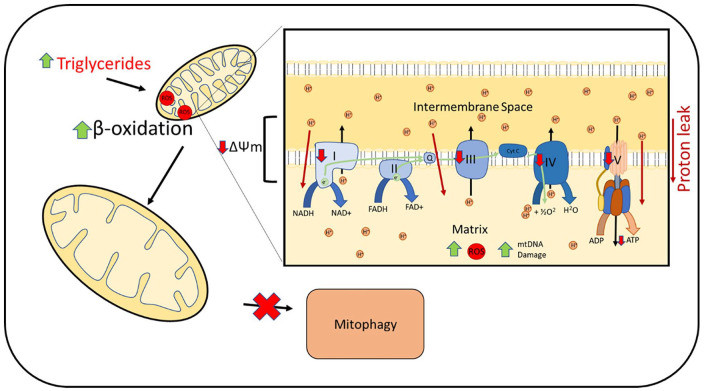
Increased hepatocyte triglycerides and fatty acids leads to upregulation of mitochondrial β-oxidation. This is associated with increased mtROS production. Increased mtROS leads to mtDNA damage and impaired complex I, III, IV and V activity. Increased proton leaks across the inner membrane is reported with impaired mitochondrial polarisation (ΔΨm). These mitochondrial defects result in impaired ATP production. Swollen mitochondria with impaired cristae are reported. Mitophagy is found to be impaired. mtDNA, mitochondrial DNA; mtROS, mitochondrial reactive oxygen species.

Increased mitochondrial metabolism is associated with an increase in mtROS production. Stimulation of mitochondrial metabolism with lipid infusion or a high-fat diet in rats resulted in a proportional increase in oxidative stress (Figure 3).^49^ Inhibition of gluconeogenesis protected against increased metabolism and oxidative damage.^49^ Models of NAFLD such as choline-deficient diet (CDD)-fed rats have increased superoxide and hydrogen peroxide production.^50–52^ Hepatocyte mitochondria from patients with non-alcoholic steatohepatitis (NASH) show increased production of hydrogen peroxide and are associated with increased evidence of tissue oxidative damage such as lipid peroxidation.^47,50,53^

In concert with increased ROS, mice fed a high-fat diet (HFD) and CDD-fed rats have reduced antioxidant defences.^49,54^ Superoxide dismutase activity and glutathione levels have been found to be significantly reduced in NAFLD and NASH patient liver tissue compared with healthy controls.^50,55^

#### Impaired oxidative phosphorylation

While being a major source of ROS, the mitochondria are also vulnerable to oxidative stress with resulting damage to mitochondrial proteins, lipids and mtDNA. CDD-fed rats have increased peroxidation of cardiolipin, an important component of the mitochondrial inner membrane, which results in reduced complex I activity.^51^ Models of obesity and NAFLD show evidence of increased mtDNA damage with impaired mtDNA repair mechanisms.^56^ NAFLD patients have an increased mtDNA mutation rate, including genes encoding complexes of the ETC with mutational burden increasing with histological disease severity.^57^ Mutations within genes encoding ETC subunits were associated with reduced expression of their corresponding messenger RNA.^57^

Oxidative mitochondrial damage leads to mitochondrial dysfunction with the development of defects in the mitochondrial ETC and oxidative phosphorylation. HFD-fed mice are found to have reduced complex IV activity with impaired respiration and mitochondrial membrane potential.^58^ CDD-fed mice had significant reduction in subunits of complex I and IV.^51,52,54^ Mitochondria from liver biopsies of patients with NASH have reduced activity in all ETC complexes compared with controls.^59^ mRNA expression of complexes I, III, IV and V were lower in NASH liver tissue compared with healthy controls.^47^ Exposure of cultured hepatocytes to palmitic acid (PA) can be used to induce hepatocyte steatosis. PA exposure is found to cause a reduction in mitochondrial polarisation.^60,61^ Several lines of evidence highlight an increase in proton leak across the inner mitochondrial membrane both in CDD-fed rats and obese mice models.^62,63^ Proton leak allows dispersal of the mitochondrial potential without ATP production, uncoupling electron transport to ATP production. Mitochondrial uncoupling is seen to increase progressively from obesity to steatosis to NASH.^47^ Increased uncoupling may be due to increased expression of uncoupling protein 2 (UCP2), which facilitates proton movement across the inner membrane. Increased UCP2 mRNA and protein expression is seen in obese mice and in methionine and choline-deficient diet rats.^50,63^ UCP2 mRNA expression was increased in liver tissue from patients with NASH compared with healthy controls.^50^

Defective electron transport as well as increased proton leak will further increase mitochondrial oxidative stress, creating a vicious cycle, further promoting mitochondrial dysfunction and impairing oxidative phosphorylation. Obese mice and CDD-fed animal models have been reported to have reduced ATP content and impaired ATP maintenance with fasting.^54,62,63^ Liver ATP generation from a fructose challenge as measured by nuclear magnetic resonance spectroscopy in patients with NASH is found to be impaired compared with healthy controls.^64^

#### Altered mitochondrial structure and dynamics

As NAFLD progresses to NASH, the mitochondria develop structural abnormalities and are reported to appear swollen with loss of cristae and paracrystalline inclusions.^53^ Megamitochondria are recognised within liver biopsies of patients with NASH.^65^ Mice fed a prolonged western-style diet high in fat, fructose and cholesterol show suppressed mitochondrial dynamics with reduced activity of transcription factors necessary for mitochondrial biogenesis as well as reduced fission and fusion proteins.^66^

#### Impaired mitophagy

The removal of damaged mitochondria through mitochondrial fission and subsequent mitophagy is a cellular protective mechanism against mitochondrial dysfunction. Proteins associated with mitophagy, including LC3II, Atg5 and Beclin 5, are reduced in hepatocytes exposed to PA with reduced Parkin expression, suggesting impaired mitophagy.^61,67^ HFD-fed mice show colocalization of mitochondria and autophagosomes after 6 weeks.^60^ However after prolonged feeding, colocalization was reduced with loss of colocalization after 24 weeks, which was associated with the development of macrosteatosis, inflammatory infiltration and fibrosis.^60^ This suggests impaired mitophagy may be an acquired defect involved in the progression into NASH.^60^

### Relevance of mitochondrial dysfunction to progression of NAFLD

#### Cell death

Mitochondrial dysfunction has an important role in determining cell fate with direct implications on disease progression. Hepatocytes exposed to PA develop increased mtROS, impaired mitochondrial polarisation and ATP production with increased cytochrome c leakage from the mitochondria. This is associated with increased expression of pro-apoptotic proteins, including caspase-3, capsase-9 and Bax and increased hepatocyte apoptosis as determined by TUNEL assay.^68^ Liver tissue from HFD-fed mice also show elevated caspase 3 and 9 with downregulation of anti-apoptotic proteins such as c-IAP.^67^ Apoptotic cell count on HFD mice liver tissue correlates to steatosis and inflammation.^60^ Liver tissue from patients with NASH show increased frequency of apoptotic cells as well as increased caspase 3 and 7 immunohistochemistry staining.^69^ Apoptosis was found to correlated with liver injury and histological severity of fibrosis and steatohepatitis.^69^

#### Promotion of inflammation by mitochondrial DNA

Mitochondrial dysfunction also plays a role in eliciting the inflammatory and profibrotic response seen in NASH. Mitochondrial dysfunction leads to the release of mtDNA. Circulating serum mtDNA is raised in patients with NASH compared with healthy controls.^70^ mtDNA correlated with liver inflammation or fibrosis on biopsy.^70^ Infusion of mitochondrial-derived damage-associated molecular patterns (DAMPs), including mtDNA into MCD-fed mice promoted hepatic stellate cell activation and promoted liver fibrosis.^70^ This effect was reduced by incubation of mitochondrial DAMPs with DNase, suggesting mtDNA is an active component of promoting fibrosis.^70^ Cultured Kupffer cells exposed to PA show increases in cytoplasmic mtDNA and are associated with activation of inflammasome NLRP3, leading to increased IL-1b production.^71^ There was evidence of mtDNA–NLRP3 interaction and complex formation.^71^ mtDNA isolated from HFD-fed mice have been shown to induce more potent proinflammatory response in Kupffer cells including increased TNF-α and IL-6 mRNA compared with wild-type mtDNA.^72^ HFD-fed-mice-derived mtDNA and circulating mtDNA from obese patients with raised ALT are found to be more potent TLR9 agonists compared with control mice and healthy subject-derived mtDNA.^73^ Oxidation of DNA increases its TLR9 agonism and increased quantities of oxidised mtDNA are reported in circulating microparticles in obese patients with deranged liver function.^73^

### Mitochondria-targeted therapies

There are currently no mitochondrially targeted treatments for NAFLD. Metformin, a commonly used biguanide antihyperglycemic, inhibits mitochondrial respiration; however, it has been found to be ineffective in treatment of NAFLD.^74,75^ However, the significance of its mitochondrial inhibition at clinically used doses is in doubt.^76^ Liraglutide has shown efficacy in clinical trials in patients with NASH.^77^ Liraglutide has been shown in PA- and LPS (lipopolysaccharide)-treated hepatocytes to ameliorate the reduction in mitochondrial respiration, polarisation and increased proton leak.^78^

Melatonin has shown some mitochondrial specific benefits in NAFLD models. Melatonin was shown to improve PA induced mtROS, mitochondrial fission and mitophagy inhibition thereby improving mitochondrial respiration and reducing cell apoptosis.^61^ R-Tf-D-LP4 is a peptide targeted to the voltage dependant anion channel 1 (VDAC1) on mitochondria.^79^ In a HFD-fed NASH mouse model, R-Tf-D-LP4 increased expression of uncoupling proteins with resulting increase in energy expenditure and was associated with reduced hepatic steatosis, ballooning and inflammation.^79^

FLINAX, a combination dietary supplement containing vitamin E, has shown in an HFD rat model to improve mitochondrial complex activity and ATP production as well as improving hepatic steatosis and reducing oxidative damage.^80^ Another animal study using HFD-fed mice reported improvements in serum transaminases and reduced hepatic steatosis after infusion with exogenous mitochondria derived from HepG2 cells.^81^ The authors also reported an improvement in hepatic mitochondrial morphology with examination under electron microscopy, improved complex IV activity and reduced hepatic oxidative stress.^81^

## Chronic hepatitis B

### Mitochondrial dysfunction in hepatitis B

The hepatitis b virus (HBV) genome consists of four overlapping open reading frames encoding proteins, including polymerase, core protein, envelope protein and X protein.^82^ HBV X protein (HBx) is a viral protein vital for efficient viral replication that has effects on cell transcription and signalling through protein–protein interactions with several transcriptional factors and components of cell-signalling pathways.^83^ HBx has been reported to localise to the mitochondria when expressed in Huh7 human hepatoma cell lines,^84–86^ the HepG2 human hepatoma cell line,^87–90^ the SMMC-7721 human hepatoma cell line,^87^ the HL-7702 human hepatocyte cell line^91^ and in primary rat hepatocytes.^88^ Colocalization is also seen when expressed in the context of the whole HBV genome in HepG2 cells.^88,90^ Direct interaction with known mitochondria-associated proteins has also been described, including VDAC3,^84,92^ cardiolipin,^93^ heat shock protein 60 (HSP60),^85^ and mitochondrial antiviral-signalling protein (MAVS).^94^

#### HBx-induced mtROS

**Figure 4.**
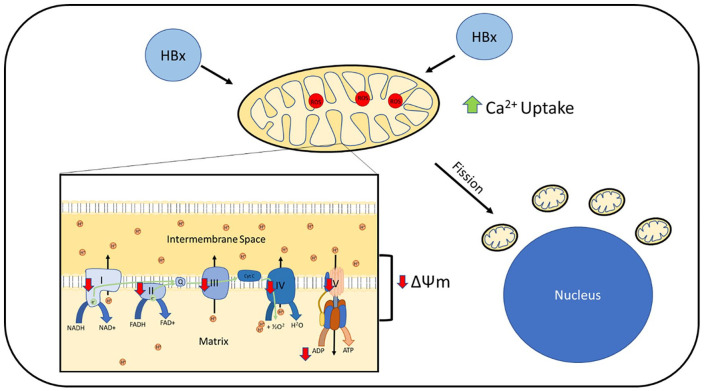
Mitochondrial dysfunction reported in hepatitis B cell models. HBx is found to localise to mitochondria in cell models. This is associated with increased mtROS with reduced activity of complexes I–V and reduced ATP production. Mitochondrial polarisation (ΔΨm) is found to be impaired. HBx expression is associated with increased mitochondrial calcium uptake. This is associated with increased mitochondrial fission. Mitochondria are found to develop a peri-nuclear distribution. HBx, hepatitis B virus X protein; mtROS, mitochondrial reactive oxygen species.

HBx expression in HepG2 and Huh7 cells was associated with increased cellular ROS (Figure 4).^92,95–97^ Increased products of lipid peroxidation were seen in HBx transgenic mice hepatocytes.^95^ Transgenic HBx-expressing mice hepatocytes were also more susceptible to ethanol and TNF-α induced increase in cellular ROS.^96^ Truncated HBx, which does not localise to the mitochondria was not associated with elevated cellular ROS, suggesting a mitochondrial source of cellular ROS.^92^ Indeed, cells of the HBx-expressing human hepatocyte cell line HL-7702 were found to have increased mtROS production and increased vulnerability to hydrogen peroxide-induced mtROS compared with controls.^91^ HBx-transfected HL-7702 cells were also found to have reduced cellular ATP compared with controls or cells expressing HBx-deficient HBV genomes.^98^

#### Impaired oxidative phosphorylation

HBx has also been reported to exert effects on mitochondrial electron transport, ROS production and ultimately, ATP production. HBx-transfected HepG2 cells have reduced activity of mitochondrial complexes I, III, IV and V.^95^ HL-7702 cells expressing HBx have reduced complex IV activity.^98^ There was preferential downregulation of mitochondrially encoded complex subunits compared with nuclear encoded subunits.^95^ Interestingly, reduced complex II activity was seen in liver biopsy specimens from patients with HBV before and during lamivudine treatment when compared with controls.^99^

Several studies report an influence of HBx on mitochondrial membrane potential. Impaired mitochondrial polarisation has been reported in HBx-expressing Huh7^84,86,100^ and HL-7702 cell lines.^98^ Similarly, isolated mouse mitochondria exposed to HBx protein have reduced mitochondrial polarisation.^93^ In opposition to this, studies in HepG2 and SMMC-7721 hepatoma cell lines expressing HBx found no change in mitochondrial membrane potential.^87,95^ However increased susceptibility to depolarisation with electron transport inhibitors was reported.^95^

Lack of consensus between studies may be due to the cell model and experimental parameters used. Clippinger *et al*.^88^ highlighted a potential role of nuclear factor (NF)-κB in modulating the mitochondrial response to HBx. HBx expression in rat hepatocytes had reduced mitochondrial depolarization compared with controls and was protected against TNF-α-induced mitochondrial depolarization. HBx expression was associated with NF-κB activation.^88^ Inhibition of NF-κB resulted in increased mitochondrial depolarization in HBx-expressing rat hepatocytes compared with controls in an MPTP-dependant manner.^88^ Therefore, HBx may have differential effects on mitochondrial polarization depending on cell stimulation and activity of other cell-signalling pathways.

In keeping with impaired electron transport and mitochondrial polarisation, HBx-transfected HL-7702 cells were also found to have reduced cellular ATP compared with controls or cells expressing HBx-deficient HBV genome.^98^

#### Altered mitochondrial structure and dynamics

Mitochondrial structure and intracellular location are affected in HBx-expressing cells. Within HepG2 and Huh7 cells, mitochondria show a predominantly fragmented structure with a perinuclear distribution.^87,97,100,101^ No morphological change was seen when cells were transfected with truncated HBx, which is unable to localise to the mitochondria.^97^ HBV has been reported to stimulate expression and activity of Drp1, while inhibiting Mfn2, thereby promoting mitochondrial fission and inhibiting fusion.^101^

#### Mitochondrial calcium homeostasis

HBx expression in HepG2 cells and HLL-7702 cells is associated with increased cytosolic calcium.^89,98^ This increase could be blocked with cyclosporin A, an inhibitor of the MPTP, suggesting HBV may manipulate intracellular mitochondrial calcium release.^89,98^ HBx is also seen to promote calcium entry from the extracellular space through store-operated calcium entry (SOCE).^102^ SOCE serves to replenish calcium stores after depletion of the endoplasmic reticulum. HBx expression is seen to increase mitochondrial calcium uptake, thereby preventing the inhibition of cell membrane calcium channels and prolonging SOCE.^102^

### Role of the mitochondria in hepatitis B pathophysiology

#### Mitochondrial immune function

MAVS is a key mitochondrial protein involved in intracellular antiviral signalling that helps transduce the detection of viral genetic material by the intracellular pattern-recognition receptors RIG-1 (retinoic acid-inducible gene I) and MDA5 (melanoma differentiation-associated protein 5) to activation of proinflammatory transcription and production of proinflammatory cytokines.^103^ HBV or HBx-transfected HepG2 had impaired activation of NF-κB and IRF-3 with impaired induction of interferon (IFN)-β transcription when stimulated with a synthetic double-stranded DNA to mimic DNA viruses or when challenged with vesicular stomatitis virus (VSV) infection.^94^ In keeping with impaired immune signalling, HBx transgenic mice hepatocytes had increased VSV replication compared with wild-type controls.^94^

HBx was found to be capable of physically interacting with MAVS protein, promoting MAVS ubiquitination, leading to a dose-dependent reduction in MAVS expression.^94^ This effect was abolished with manipulation of the HBx mitochondrial targeting sequence.^94^ HBx was found to impair MAVS interaction with downstream signalling molecules by inducing increased linear ubiquitination through Parkin-dependent activation of linear ubiquitin assembly complex (LUBAC).^104^ Clinical evidence for a MAVS defect in patients with HBV can be seen in a reported significant reduction in MAVS expression in HBV-related hepatocellular carcinoma (HCC) tissue compared with HCC unrelated to HBV and healthy controls.^94^

Additionally, mitochondrial dysfunction in HBV-specific CD8^+^ T cells has been reported in chronic hepatitis B compared with those with resolution or healthy controls.^105^ Reduced mitochondrial gene expression, impaired mitochondrial polarisation and increased mtROS production were described.^105^ Treatment of exhausted HBV-specific T cells with a mitochondrially targeted antioxidant, alongside HBV peptide stimulation, reduced HBV peptide-induced mitochondrial depolarization, reduced T cell apoptosis and improved cytokine production.^105^

#### Viral replication

Intracellular calcium levels can act an important cell-signalling mechanism and can be manipulated by viruses to promote viral replication. Inhibition of extracellular calcium entry and SOCE specifically impaired HBV viral replication.^102^ HBV replication within HepG2 cells and primary rat hepatocytes was reduced when treated with a calcium chelator.^106,107^ Impairing mitochondrial calcium release with a mitochondrial sodium–calcium pump inhibitor or MPTP inhibitor reduced viral replication, suggesting a mitochondrial source.^107^ HBV manipulates the cell cycle to promote replication in a calcium dependant manner.^108^ HBV cell cycle changes are also found to be inhibited by blocking the MPTP.^106^ HBV polymerase activity was impaired by both calcium chelation and MPTP inhibition.^106^ These results suggest HBV may manipulate mitochondrial calcium storage and release to promote viral replication *via* HBV polymerase.

#### Apoptosis

Mitochondrial dysfunction is a known trigger for programmed cell death. HBx-expressing Huh7 cells were associated with increased apoptosis, which was dependant on HBx trafficking to the mitochondria and was associated with mitochondrial depolarization.^86^ Inhibition of the MPTP as well as ROS scavengers prevented HBx-associated increase in apoptosis.^86^ Similarly hepatocytes from HBx transgenic mice were more susceptible to apoptosis when challenged with alcohol or TNF-α.^96^ HBx expression in HL-7702 cells promoted mitochondrial translocation of the pro-apoptotic protein Bax after challenge with hydrogen peroxide.^98^ This was prevented by inhibition of the MPTP.^98^

Some studies suggest a potential anti-apoptotic effect of HBV. HBV expression in HepAD38 cells was associated with stimulation of mitophagy in a Parkin-dependant manner.^101^ Inhibition of Parkin led to promotion of apoptosis in HBV-expressing cells, suggesting stimulation of mitophagy protects against apoptosis allowing viral replication to persist.^101^

### Mitochondrially targeted therapies

There are no mitochondrially targeted therapies currently under investigation; however, in view of the published evidence, potential targets could include mitochondrially targeted antioxidants or therapies targeted at mitochondrial calcium regulation or MAVS.

## Hepatocellular carcinoma

### Mitochondrial dysfunction in hepatocellular carcinoma

#### Impaired oxidative phosphorylation

**Figure 5.**
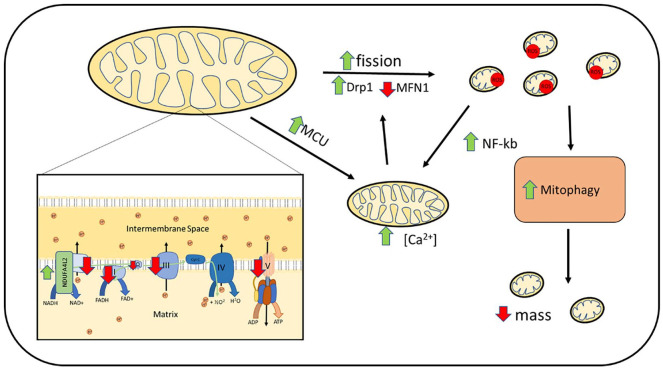
Mitochondrial dysfunction identified in HCC cell culture models and human tissue. Reduced complex I, II, III and V function is reported. Expression of complex I subunit, NDUFA4L2 is upregulated and results in impaired complex I activity. Drp1 is upregulated and MFN1 is downregulated in HCC tissue leading to promotion of mitochondrial fission. This is associated with increased mitochondrial ROS production leading to activation of NF-κb signalling and subsequent increase in mitochondrial calcium concentration. Increased mitochondrial calcium leads to activation of NFATC2 and c-Myc and further promotion of Drp1 expression and mitochondrial fission. Upregulation of MCU is reported which contributes to increased mitochondrial calcium uptake. Mitophagy is found to be promoted and is associated with reduced mitochondrial mass. Drp1, dynamin-related protein; HCC, hepatocellular carcinoma; MCU, mitochondrial calcium uniporter; MFN1, mitofusin 1; NDUFA4L2, NADH dehydrogenase 1 α-subcomplex-4-like 2; NFATC2, nuclear factor of activated T cells cytoplasmic 2; NF-κb, nuclear factor κ-light-chain-enhancer of activated B cells; ROS, reactive oxygen species.

HCC tissue is found to have reduced mitochondrial mass with reduced complex II, III and V expression.^109–111^ Expression of complex I subunit, NADH dehydrogenase 1 α-subcomplex-4-like 2 (NDUFA4L2), reduces complex I activity (Figure 5). NDUFA4L2 expression is upregulated in HCC tissue compared with nontumorous tissue and is associated with poorer overall survival.^112^ Prenyl diphosphate synthase subunit 2 (PDSS2) is an enzyme involved in the production of coenzyme Q10, which plays a critical role in the ETC. PDSS2 is downregulated in HCC tissue and associated with poorer overall survival.^113^ PDSS2 knockdown in HCC cell lines impaired complex I activity and oxidative phosphorylation.^113^

#### Altered mitochondrial structure and dynamics

Several structural mitochondrial defects are reported in HCC tissue and cell lines. Electron microscopy of human HCC tissue reveals abnormal mitochondrial architecture with reduced cristae.^109^ Mitochondria within human HCC tissue are shorter in length compared to paired nontumorous tissue.^114^ Several studies report upregulation of mitochondrial fission proteins (Drp1) and downregulation of mitochondrial fusion proteins (MFN1) in both human HCC tissue and HCC cell lines.^114–116^ Upregulated mitochondrial fission was found to be associated with more advanced tumour staging, vascular invasion and poorer outcome in HCC patients.^115,117^ Mitochondrial fission was further upregulated in human metastatic HCC tissue compared to primary lesions.^117^ Increased fission was associated with increased mtROS in HCC cell lines.^114^

#### Altered mitochondrial calcium homeostasis

Mitochondrial calcium signalling is found to be dysregulated within HCC tissue. The MCU and its regulatory proteins subunit (MCUR1) are upregulated in HCC tissue compared with matched normal tissue.^29,118,119^ Upregulation of MCU and MCUR1 in HCC cell lines is associated with increased mitochondrial calcium.^29,119^ Increased expression of MCU or MCUR1 was associated with poorer overall survival and recurrence-free survival.^29,119^ Additionally, mtDNA is found to be affected with reduced copy number and increased mutational burden.^109,110^

Mitochondrial dynamics and calcium signalling in HCC have been proposed to form a positive feedback loop. Increased mtROS induced by increased mitochondrial fission activates NF-κB, resulting in STIM1-mediated promotion of SOCE.^120^ Increased calcium entry induces activation of transcription factors NFATC2 and c-Myc, leading to expression of mitochondrial fission proteins Drp1 and FIS1 (figure 5).^120^

### Role of mitochondria in HCC pathogenesis

#### Tumour survival

Upregulation of mitochondrial fission in HCC cell lines was protective against mitochondrial depolarization and promoted cell autophagy, thereby reducing mitochondria-mediated apoptosis.^114^ Mitophagy is further enhanced by upregulation of mitophagy receptor FUNDC1 further inhibiting mitochondria-mediated apoptosis.^110^ Drp1 overexpression increased mtROS-mediated activation of NF-κB, which promotes p53 degradation, thereby inhibiting p53-mediated apoptosis.^114^ PGAM5 is a mitochondrial serine/threonine phosphatase that is upregulated in HCC tissue.^121^ PGAM5 interacts with anti-apoptotic protein Bcl-XL, thereby preventing its degradation and inhibiting apoptosis.^121^ Additionally, increased MCUR1 expression was associated with increased expression of anti-apoptotic protein Bcl-2.^119^

Increased MCUR1 expression promotes the expression of cell cycle proteins promoting tumour growth.^119^ Due to their rapid growth, tumours commonly outgrow their blood supply leading to tissue hypoxia. Reduced complex I activity *via* upregulation of NDUFA4L2 is protective against hypoxia-induced ROS, mitochondrial depolarization and subsequent apoptosis, thereby promoting tumour survival.^112^

Tumour associated macrophages (TAMs) promote cancer immune evasion and are associated with worse overall survival and recurrence-free survival.^116^ Increased mitochondrial fission promotes mtROS and cytoplasmic release of mtDNA.^122^ mtDNA and HMGB1 can stimulate TLR9 both individually and in complex.^122^ TLR9 activates NF-κB signalling, promoting the expression of CCL2 and increasing TAM recruitment.^116^ Therefore, changes in mitochondrial dynamics influence cell signalling to promote TAM infiltration and tumour immune evasion.

#### Promotion of metastasis

Upregulation of mitochondrial fission by increased Drp1 expression was associated with an increased number of intrahepatic and distant metastasis in xenograft nude mice models.^117^ MFN1 downregulation was associated with increased vascular invasion and poorer prognosis in HCC tissue.^115^ Transition from an endothelial state to a mesenchymal state promotes tumour cell mobility and invasiveness, which is required for tumour metastasis. MFN1 inhibits epithelial-to-mesenchymal transition *via* inhibition of key transcription factors such as Snail.^115^ Drp1 upregulation or MFN1 downregulation promote lamellipodia formation in a calcium-dependent manner thereby promoting metastasis.^117^

Upregulation of MCU increases mitochondrial calcium, leading to mtROS-dependant activation of c-Jun N-terminal kinase (JNK). JNK activation upregulates matrix metalloproteinase-2 and promigratory cell changes, including lamellipodia formation.^29^ MCUR1 upregulation promotes epithelial-to-mesenchymal transition in HCC cells through activation of Snail *via* a mtROS/Nrf2/Notch1-mediated pathway.^118^ Increased MCU or MCUR1 expression in mouse models displayed higher intrahepatic and distal metastasis.^29,118^

### Mitochondria-targeted treatments

There are currently no clinical trials evaluating mitochondrially targeting treatments in HCC. However, several lines of evidence suggest the potential of modulating mitochondrial function. Sorafenib, a multi-kinase inhibitor, is currently first-line systemic chemotherapy for HCC. Several studies have highlighted its inhibitory effects on mitochondrial function. Sorafenib is reported to inhibit complex I and III, reduce mitochondrial polarisation and promote mitochondrial proton leak with resulting reduction in mitochondrial respiration and ATP production.^123,124^ Impaired ATP production leads to AMPK activation and antiproliferative cell signalling independent of its kinase inhibitor function.^123^ It has also been shown to induce mtROS and promote mitochondrial swelling, cytochrome c release and apoptosis.^125^ Similarly, metformin, a known inhibitor of complex I and AMPK activator, has been found to be associated with a reduced risk of developing HCC in patients with diabetes.^126^ In HCC cell culture and xenograft models metformin is associated with reduced tumour proliferation and increased apoptosis.^126,127^

Novel mitochondrial therapies are being investigated. A mitochondrial VDAC-targeted peptide, R-Tf-D-LP4, was evaluated in HCC cell lines and found to inhibit binding of anti-apoptotic proteins, inhibit mitochondrial respiration and promote apoptosis.^128^ In xenograft models and in DEN (diethylnitrosamine)-induced mouse models, R-Tf-D-LP4 was found to inhibit and impair tumour growth, promote apoptosis and reduce inflammatory infiltration.^128^ An interesting study into the effect of untargeted and mitochondrially targeted antioxidants in HCC models shows the importance of understanding the role of mitochondria in tumorigenesis.^129^ Mitochondrially targeted antioxidants were found to promote the development and growth of HCC in DEN-induced HCC models, whereas untargeted antioxidants were found to be beneficial.^129^ This highlights the importance of fully elucidating the role of mitochondrially generated ROS and mitochondrial signalling in regulation of apoptosis and cell proliferation.^129^

## Conclusion

**Figure 6.**
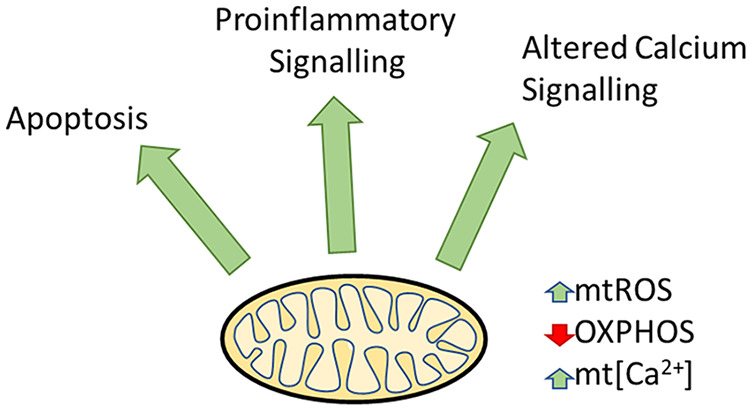
Common downstream effects of mitochondrial dysfunction in chronic liver disease, including alterations in apoptosis, inflammatory signalling and calcium signalling. Common mitochondrial features include increased mtROS, reduced OXPHOS and increased mt[Ca^2+^] mtROS, mitochondrial reactive oxygen species; mt[Ca^2+^], mitochondrial calcium concentration; OXPHOS, oxidative phosphorylation.

Mitochondrial defects are well recognised within human tissue and disease models of ARLD, NAFLD, chronic HBV and HCC. Despite significant differences between pathophysiology, common mitochondrial defects emerge, including increased mtROS and impaired oxidative phosphorylation. This leads to alterations in hepatocyte cell metabolism, ROS signalling, cell apoptosis and inflammatory signalling (Figure 6). Currently therapies that target mitochondrial dysfunction are lacking. Further research to fully elucidate the contribution of mitochondrial dysfunction in the pathology of these common liver diseases as well as new methods to manipulate mitochondrial function is required to exploit this potentially powerful therapeutic target.
